# Acousto-Optic Comb Interrogation System for Random Fiber Grating Sensors with Sub-nm Resolution

**DOI:** 10.3390/s21123967

**Published:** 2021-06-08

**Authors:** Dragos A. Poiana, Jose A. Garcia-Souto, Xiaoyi Bao

**Affiliations:** 1 Physics Department, University of Ottawa, Ottawa, ON K1N 6N5, Canada; Xiaoyi.Bao@uottawa.ca; 2 Sensors and Instrumentation Techniques Research Group, Electronics Technology Department, University Carlos III de Madrid, Leganes, 28911 Madrid, Spain; jsouto@ing.uc3m.es

**Keywords:** self-heterodyne comb, random fiber grating, FBG, ultrasound

## Abstract

The broad-frequency response and nanometer-range displacements of ultrasound detection are essential for the characterization of small cracks, structural health monitoring and non-destructive evaluation. Those perturbations are generated at sub-nano-strain to nano-strain levels. This corresponds to the sub-nm level and, therefore, to about 0.1% of wavelength change at 1550 nm, making it difficult to detect them by conventional interferometric techniques. In this paper, we propose a demodulation system to read the random fiber grating spectrum using a self-heterodyne acousto-optic frequency comb. The system uses a self-heterodyne approach to extract phase and amplitude modulated signals to detect surface acoustic waves with sub-nanometer amplitudes in the frequency domain. The method can detect acoustic frequencies of 1 MHz and the associated displacement. The system is calibrated via phase detection with a heterodyne interferometer, which has a limited frequency response of up to 200 kHz. The goal is to achieve sub-nanometer strain detection at MHz frequency with random fiber gratings.

## 1. Introduction

Ultrasound measurements are very important in several fields such as structural health monitoring, crack detection and non-destructive evaluation [1]. The broad-frequency response with small displacement (nanometers) of ultrasound detection, needed for crack characterization, is associated with small displacements, which can be as low as sub-nano-strains. This makes the detection system very challenging, and this process requires MHz frequency response where ultrasound superficial mechanical waves usually occur.

Fiber-based sensors have been used for the measurement of ultrasound waves from point sensors to distributed sensors [2]. Their exceptional properties of electromagnetic emission immunity, chemical immunity and physical properties make them reliable sensors to detect sub-micrometer amplitude ultrasound waves. They are based on measuring one or more properties of the fiber that has a significant dependence on the strain applied to it.

Functionalized fiber sensors have been developed to make the fiber more sensitive to change, either based on doping with a compound or based on changing its geometry or internal structure. Fiber Bragg gratings (FBGs) [3,4] are optical fiber sensors that have been functionalized to have a periodic diffractive pattern inscribed in their core. This makes them behave as optical filters. The central wavelength, or Bragg Wavelength is dependent on the strain and temperature applied to the fiber.

Random fiber gratings [5] are devices that can be used to detect ultrasound and temperature. However, their spectrum is degenerated compared to a uniform FBG device, as the periodical pattern inscribed is random. Therefore, the back-reflection response of the sensor with respect to the wavelength is not Gaussian-shaped as in the case of uniform FBGs. At the same time, it spreads over a broader wavelength interval that is usually in the range of 200-nm width or more. The approximative wavelength shift sensitivity with respect to mechanical strain applied is 1.2 pm/με as shown in [6]. The dependence of the Bragg wavelength with temperature is usually 10 pm/K. However, those changes occur at low frequencies’ measurement due to slow response of temperature, and have little impact to our high-frequency (sub-MHz) mechanical perturbations.

This random period behavior is linked to a random spectrum, and therefore, the back reflection of the sensor is a wavelength-dependent random function. However, some other deterministic properties remain true. For example, in random fiber gratings, the spectrum shifts linearly with respect to the strain applied [7] as in the case of fiber Bragg grating devices. It can be seen that for the same strain applied to the random fiber grating sensor, a correlation peak shift occurs, and it is a fixed and deterministic quantity that can be seen with an optical spectrum analyzer (OSA). Due to the slow time response of the wavelength sweep of the optical spectrum analyzer (in the ms range), the translation from wavelength to a digitally displayed value occurs at a very slow rate, making this approach unsuitable for measuring the strain caused by ultrasound signals (at hundreds of kHz to MHz).

Optical frequency combs [8,9] have been used widely since their discovery for spectroscopy [10,11], vibration measurements [12] and high-precision metrology systems. They are sources that have a broadband spectral range over multiple equally spaced discrete modes that behave coherently. Therefore, the information of the wavelength is obtained in real-time with a refreshing frequency in the range of hundreds of MHz. Hence, the readout of fast-speed change at ns speed in the wavelength domain can be realized.

Dual combs represent multi-heterodyne of two optical spectrums for obtaining an electrical set of harmonics. This tool constitutes a worthy example that involves the readout of a wavelength range from the resulting RF beats. Their structure is similar to the optical frequency combs, that is, a set of wavelengths that are distributed along a specified wavelength range; however, the spacing between homologue tones from each source is different and this is what allows the injective mapping from the optical to electrical domain. This is very useful for spectroscopy and other applications [13,14,15,16,17]

The dual comb structure allows several degrees of freedom in the harmonic distribution in electrical domain. However, it involves higher complexity than the single optical frequency comb and this fact can diminish its usability in particular contexts where smaller setups are required [18].

In this study, the main objective was to use the acousto-optic comb readout to interrogate random fiber gratings and to demonstrate high-frequency and high-sensitivity measurements. As a secondary objective, we aimed to compare the performance of the uniform FBG sensors and the random fiber gratings for vibration measurements [19]. Comparison and calibration of the sensor were performed using an interferometer [12].

For those goals, we propose a readout system based on acousto-optic optical frequency comb [20,21,22] to read random fiber grating sensors inscribed by a “plane-by-plane” writing technique. These sensors can be used for strain sensing [23]. We demonstrated high SNR of the acousto-optic combs compared to electro-optical frequency combs and used lock-in amplification techniques to recover the strain applied to the random fiber sensor. Finally, we calibrated the system via an interferometer to quantify the sub-nanometer amplitude values based on the sideband-ratio method to recover the strain applied, which was compared with uniform FBG.

## 2. Materials and Methods

### 2.1. Main System

The main system uses an acousto-optic comb interrogator to read the optical sensor that can be either a random fiber grating or a Uniform Fiber Bragg grating.

The interrogator generates an acousto-optic optical frequency comb, which is a finite set of optical harmonics that is injected into the random fiber grating sensor using a circulator. If a mechanical wave perturbation is applied to the grating, the phase and amplitude of each optical tone of the interrogating comb change, and therefore the information of the vibration can be read from the electrically generated photo-detected comb.

**Figure 1:**
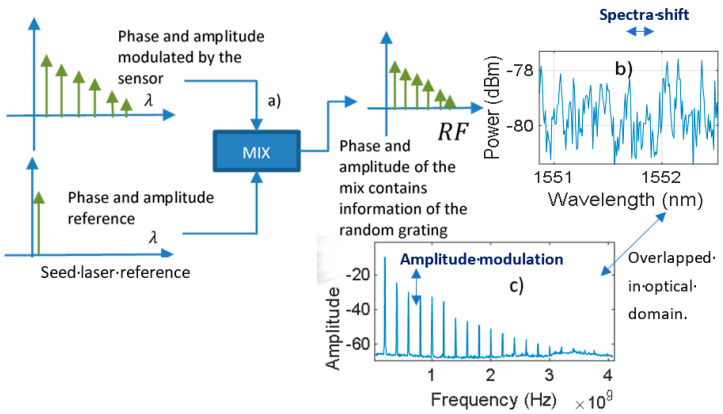
Demodulation: (**a**) mixing process that transduces the vibration measurement from the modulated optic spectra to RF; (**b**) optical reflection response of the random fiber sensor, which modifies the optical comb parameters; (**c**) spectra of the self-heterodyne comb.

From the nature of the interferometric self-heterodyne comb architecture, the variations in the i-th optical amplitude are translated into changes in the i-th amplitude of the detected RF comb. In Figure 1a, we can observe how the acoustic comb is mixed with the seed laser after traveling through the sensor. This process constitutes the self-heterodyne mix in the RF domain and the spaced frequency is equal to the optical spacing of the acousto-optic comb. As the seed laser acts as a stable reference, any variation over the acoustic comb amplitudes and phases is translated into an RF multiheterodyne signal.

On the left part of Figure 1a we can see the optical domain, and on the right part we can see how any phase and amplitude change can be read with the electrical spectra of the self-heterodyne comb [24]. Therefore, in Figure 1b, the spectrum of the sensor modulates the optical self-heterodyne comb, and the result is downshifted from optical frequency to RF frequency as shown in Figure 1c. This is because if a horizontal displacement occurs on the sensor spectrum (Figure 1b spectra shift) a variation in amplitude of the RF comb can be seen (Figure 1c amplitude modulation).

**Figure 2:**
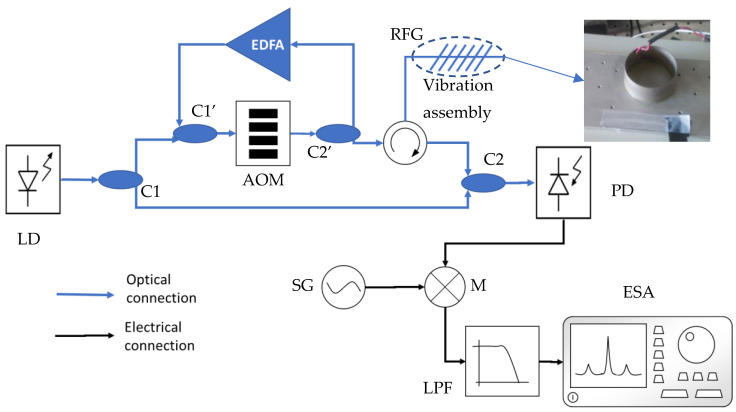
Self-heterodyne comb readout system together with the vibration assembly view. C1, C1’, C2, C2’: All couplers are 50:50 couplers. EDFA: erbium-doped fiber amplifier, AOM: acousto-optic modulator driven with a sinusoidal 200 MHz signal, LD: laser diode PD: photo-detector, LPF: low pass filter, ESA: electrical spectrum analyzer, SG: signal generator, M: mixer, RFG: random fiber grating.

The implementation setup can be seen in Figure 2. We used a high-quality continuous-wave laser for the main system. The beam is split utilizing a directional coupler C1, and the acousto-optic comb is generated with an acousto-optic modulator (AOM) and amplification feedback with an EDFA and two 50:50 couplers: couplers C1’ and C2’. The optical comb signal is injected into the random fiber grating through a circulator and the backward reflection is mixed with the seed laser with C2 (output coupler Figure 1a). Finally, the optical output is photo-detected with a fast PD, the response of which in the frequency domain is a comb similar to Figure 1c.

The spectrum of the self-heterodyne comb [24] is placed at 1551.8 nm, and it has an interline space of 1.6 pm. It is modulated by the sensor exposed to ultrasound and afterward mixed with the phase and amplitude optical reference. Consequently, any change in the reflection response of the random fiber grating can be read from the radio frequency (RF) domain of the acousto-optic comb.

The acousto-optic combs can achieve very high SNR, and at the same time they are simple to set and build. In our case, we can obtain a noise floor of −60 dBc on 4 GHz of bandwidth.

The acousto-optic combs are suitable for applications in which the amplitude of the modes is highly attenuated as in the case of random grating back reflection; in our case, the attenuation of the carriers is 30 dB. Therefore, a high SNR of the input source is needed.

The input electric field of the circulator (circulator of Figure 2) can be expressed as a sum of each optical harmonic (Equation (1)):

(1) $$E_{comb}\left(t,z_{c}\right)=E_{0}\overset{N}{\underset{a=1}{\sum }}A_{a}e^{j\;\left((\omega_{0}+a\Delta \omega_{AOM})\;t-\frac{2\pi n}{\lambda +a\Delta \lambda }\;\left(z_{c}+\left(a-1\right)\Delta L\right)+\phi_{a}\right)}$$

where $E_{0}$ represents the electric field in the C1’ coupler input, and $A_{a}$ represents an attenuation factor of the a-th optical tone, dependent on the gain of the EDFA amplifier and the insertion losses of couplers C1’ and C2’ and the AOM with respect to the wavelength. $\Delta \omega_{AOM}\;$represents the frequency shift produced by the AOM. Δλ represents the wavelength shift of the light caused by the AOM, and ΔL is the delayed fiber length that the light travels along the feedback loop. n is the refractive index of the fiber, λ is the wavelength of the seed laser and $\phi_{a}$ is the initial phase. Finally, $z_{c}$ is the particular space coordinate measured along with the optical fiber where the electric field is evaluated, and $\omega_{0}$ is the frequency of the optical carrier. N refers to the maximum number of harmonics on the acousto-optic comb. The process of injecting the light to a random fiber grating on which we apply vibration strain signals leads to a modulation in the carrier signals expressed in Equation (1). Therefore, the amplitude is modulated by the reflection response of the random fiber grating sensor, and similarly, the dispersion profile of the random fiber grating sensor modulates the phase of the carriers. Notably, each carrier is modulated with different characteristics as the dispersion profile and the absorption profile are wavelength-dependent functions and the carriers are placed at different wavelengths spaced at a frequency equal to the AOM driving signal ($\Delta \omega_{AOM}\;$= 200 MHz).

The mix of Equation (1) with the second arm of the interferometer leads to a heterodyne interferogram for each pair of optical modes, so the overall output is a multiheterodyne interferogram. The resulting intensity is in the form of Equation (2):

(2) $$I\left(z_{c},t\right)\propto \overset{N}{\underset{a=1}{\sum }}\begin{array}{c} 2E_{0}^{2}\;A_{a}\left(t,\lambda +a\Delta \lambda \right)cos(a\Delta \omega_{AOM}t+\frac{2\pi n}{\lambda +a\Delta \lambda }\;\left(z_{c}+\left(a-1\right)\Delta L\right)-\frac{2\pi n}{\lambda }z_{1}\\ +\phi_{a}\left(t,\lambda +a\Delta \lambda \right)) \end{array}$$

where $A_{a}\left(t,\lambda +a\Delta \lambda \right)\;$is a function that represents the attenuation of each frequency component due to the amplitude modulation caused by the random fiber grating sensor. It is dependent on the wavelength because the reflection of the random fiber grating is a wavelength-dependent function. Simultaneously, it is a time-dependent function as the correlation peak of the reflection response of the random fiber grating changes with time if the vibration is applied to the random fiber grating. $\phi_{a}\left(t,\lambda +a\Delta \lambda \right)$ denotes a function that represents phase variation of each optical mode electrical field due to the dispersion profile of a random fiber grating sensor. Therefore, if the lock-in technique is used, both$\;A_{a}\left(t,\lambda +a\Delta \lambda \right)$ and $\phi_{a}\left(t,\lambda +a\Delta \lambda \right)\;$can be extracted, and this demonstrates that the vibration of the random fiber grating can be measured if a small-signal approach is assumed. To ensure this, the amplitude of the vibration or acoustic waves should be very small compared to the random period of the random fiber grating. $z_{1}$ is the space coordinate measured along with the reference optical fiber of the interferometer.

**Figure 3:**
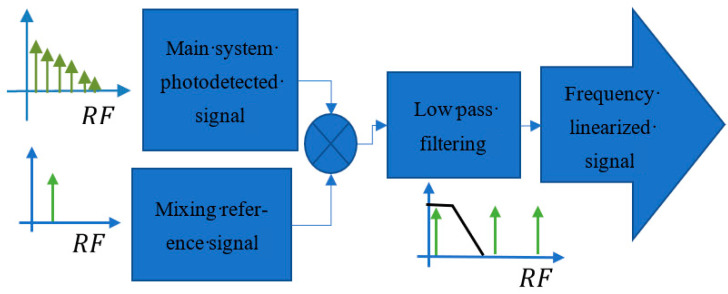
Electronic demodulation through analog electronics applied to RF output of the main system.

The small-signal approach enables us to measure with linearity in small regions of wavelength, translating the vibration to linear amplitude modulation of the comb lines. The photo-detected signal of coupler C2 is mixed with a reference signal and afterward lowpass filtered as shown in Figure 3. The process enables independent readout for each line of the comb as each one is placed at an integer multiple of the acousto-optic frequency.

The mixing reference frequency should be the same as the targeted locking frequency. If the first harmonic is being measured, $a\Delta \omega_{AOM}\;$of the frequency should be chosen for a = 1. The result is a linearization with respect to the frequency as the beat is moved to DC and not to a specific carrier.

### 2.2. Calibration System

For quantifying the strain applied on the random fiber grating, an auxiliary interferometer was built to obtain an absolute value of displacement of the mechanical acoustic wave applied to the sensing part. The interferometer uses the same seed laser with a 200 MHz offset frequency that is provided by an acousto-optic modulator for the heterodyne case. The noise floor level is −75 dBm or lower over a 200 kHz measurement bandwidth. The demodulation algorithm for the interferometer is described in [19].

It consists of a sideband suppression ratio. When we modulate in phase any heterodyne signal of an interferometer, we generate sidebands around the heterodyne carrier. The ratio between the sidebands and the heterodyne carrier is an indicator of the extent of the modulating depth applied and therefore the amplitude of the modulation signal. We can assume that the phase modulation of the heterodyne interferometer is of the form of Equation (3), where $\omega_{carrier}$ is the frequency of the carrier generated by the acousto-optic displacement and $\varphi_{modulation}\sin(\omega_{modulation}t)$ is the strain induced phase modulation of amplitude $\varphi_{modulation}$ and frequency $\omega_{modulation}$.

(3) $$\varphi_{0}=\omega_{carrier}t+\varphi_{modulation}\sin(\omega_{modulation}t)$$

$\varphi_{modulation}\;$can be easily extracted from the ratio of the carrier and the sidebands in the frequency domain.

Once this value is extracted, the absolute displacement$\;z_{m}$ from Equation (4) can be obtained, where $n_{0}$ is the refractive index and λ is the optical wavelength of the laser that feeds the interferometer.

(4) $$\varphi_{modulation}=2\pi n_{0}z_{m}/\lambda$$

The displacement produced by the vibration is related to the strain by the effective length on which the vibration $L_{eff}$ is applied according to Equation (5).

(5) $$\varepsilon =\frac{\Delta L}{L_{eff}}=\frac{z_{m}}{L_{eff}}$$

Good results were obtained because a narrow linewidth laser (<100 Hz) was used, and a lookup table was employed, leading to higher SNR, and therefore a better resolution of sub-nanometer displacement was achieved. The random fiber grating and the interferometer sensing part were placed 5 cm away from a piezo-stretcher (PZT of Figure 2) and attached to an aluminum plate to ensure that the surface acoustic waves generated by the piezoelectric device were directed towards the optical fiber. Sinusoidal signals of different amplitudes and frequencies were applied to the PZT, and the output of both systems, the heterodyne interferometer and the random fiber grating interrogating system, was recorded.

The seed laser used for both the calibration and the main system is a Koheras Adjustic E15 that provides a low phase noise of 100 dBc/Hz. The demodulating process used analog multipliers of 800 MHz bandwidth and lowpass filtering at 11 MHz for amplitude lock-in recovery of each tested harmonic.

## 3. Experimental Results

### 3.1. Calibration

**Figure 4:**
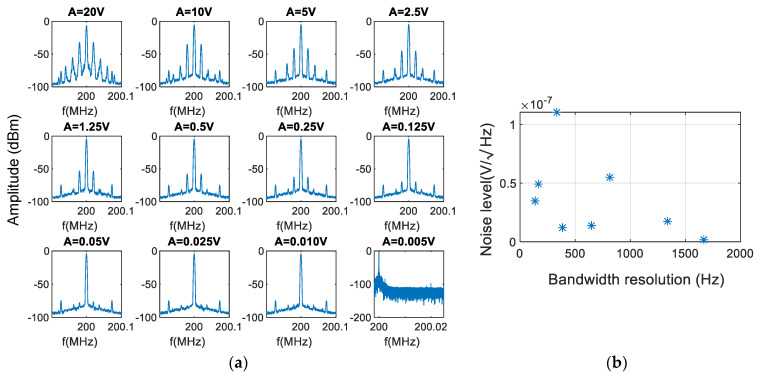
Calibrating interferometer: (**a**) response to different stimuli at 20 kHz generates a sideband modulation over the carrier. Ratios between the carrier and the first-order sidebands allow absolute calibration (A is the amplitude of the excitation voltage applied), (**b**) noise floor level achieved for different bandwidth resolutions.

In Figure 4a, several calibration traces are shown for the auxiliary interferometer used for calibration. From 20 V to 5 mV, the ratios between the carrier and the first-order sidebands were from 30 dB in the case of 20 V excitation to 95 dB in the case of 5 mV excitation. As stated, the reference levels of strain amplitude can be associated with an attenuation between the central mode and the first sideband (intermodal attenuation) of the calibrating interferometer, and those levels of strain can be associated with the measurements made by the main system for calibration.

In Figure 4a, we can observe the calibration traces for each one of the strain levels for the auxiliary interferometer (from A = 20 V to A = 0.005 V trace). This accuracy is achieved at 25 kHz bandwidth, and it is read in the frequency domain. From the case of the minimum signal applied to the transducer (A = 0.005 V trace), a white noise level of $\frac{50\mathrm{nV}}{\sqrt{\mathrm{Hz}}}$ for a measurement bandwidth of 25 kHz can be easily extracted. In Figure 4b we can observe the noise floor level with respect to the bandwidth resolution of the frequency domain.

To obtain the absolute strain amplitude, we calculated the ratio between the Bessel function of the first kind and order zero and the Bessel function of the first kind of order one ($J_{i}\left(\varphi_{modulation}\right)$/$J_{i+1}\left(\varphi_{modulation}\right)$).

Several traces were registered in the frequency domain, and by using a lookup table of the Bessel functions of the first kind we converted this ratio to depth modulation. We then transformed it to strain applied over the measuring arm [19].

For example, if the amplitude of the sideband harmonics is half of the carrier, thus $J_{1}\left(d\right)/J_{0}\left(d\right)=0.5$ or an equivalent 6 dB attenuation in RF power scale, the depth modulation $\varphi_{modulation}$ is 0.9 rad. This corresponds to an equivalent average displacement of 148 ±3nm using Equation (4).

**Figure 5:**
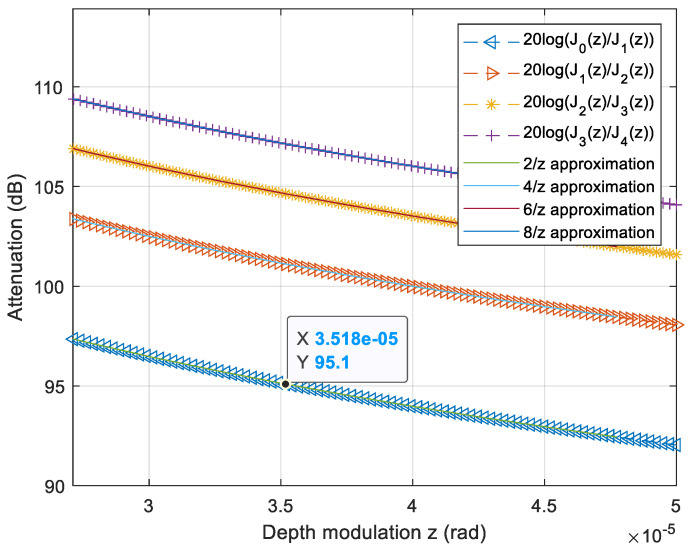
Values of the ratio between consecutive Bessel order for the interferometric calibration $J_{i}\left(\varphi_{modulation}\right)$/$J_{i+1}\left(\varphi_{modulation}\right)$. Marker at 95 dB of attenuation (*y*-axis) between the zero and the first order of Bessel functions corresponding to lower detectable amplitude (A = 5 mV from Figure 4a). The modulation depth $\varphi_{modulation}$ is shown in the *x*-axis. The first term of Taylor approximation (hyperbolas) is also depicted for each function.

The result for the minimum achievable displacement is shown in the plot of Figure 5, and the best resolution is about 35 μrad (from the data tip) of optical phase difference for 5 mV of electrical excitation over the PZT obtained at 95 dB attenuation in the frequency domain trace. This resolution is dependent on the bandwidth of the FFT measurement. Therefore, at 200 kHz, the attenuation between $J_{0}$ and $J_{1}$ corresponds to 72 dB of power attenuation as shown in the A = 0.025 V plot of Figure 4a, which translates to a 0.5 mrad optical phase depth modulation ($\varphi_{modulation}$), almost one order worse than the one obtained for the best case. This relationship is not linear because the tendency of the function plotted in Figure 5 is hyperbolic for *z* near 0.

### 3.2. The Main System

When the vibration is applied to the random fiber grating, the phase and amplitude of the optical harmonics of the self-heterodyne comb are modulated accordingly. Consequently, that information can be read directly through the photo-detected interferogram of the main system. The physical arrangement is as shown in Figure 2. The PZT was joined with a metal plane, and the surface acoustic waves were measured with the optical fiber that is stuck to the surface of the plane. The main system was also tested with uniform FBG to compare it with the random fiber grating sensor. In the case of uniform FBG, the wavelength of the laser must be carefully aligned with the slope of the back-reflection of the sensor, while in the random fiber grating case, the laser always lies inside the bandwidth of the random fiber grating back-reflection, and therefore no aligning process is needed.

The minimum detectable amplitude is 35 μrad calibrated with heterodyne interferometer zero and first orders of the Bessel functions. Those amplitudes correspond to A = 5 mV of Figure 4a.

**Figure 6:**
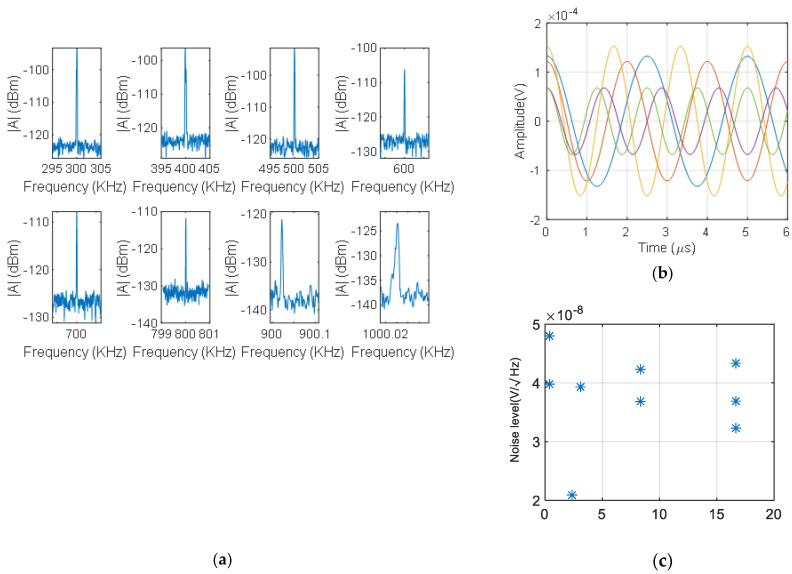
Measured output signals at different mechanical excitation frequencies over the random fiber grating: (**a**) the frequency domain of the signals, (**b**) time domain of the surface acoustic waves, (**c**) noise floor level for different bandwidth resolutions.

In Figure 6a we can observe the response of the sensor to vibrations at different frequencies. Measurements are shown in Figure 6a for several mechanical frequencies (from 300 kHz to 1 MHz). In Figure 6b we can observe the time domain traces for the same signals obtained from the inverse Fourier transform of each in Figure 6a. In Figure 6c we can observe the noise floor level with respect to the bandwidth resolution. It can be seen that, if the resolution of the frequency domain is improved, we can achieve a noise floor level of approximately$\;40\;\mathrm{nV}/\sqrt{\mathrm{Hz}}$ on average in the frequency domain trace that is equivalent to 70 μV.

Those results demonstrate the hypothesis of linearity between the input mechanical signal and the output detection signal when a random fiber grating is used. Therefore, for a particular input frequency, the output signal has the same frequency and an amplitude that is proportional to the modulation of the random fiber grating sensor.

The noise floor of each measurement can be seen in Figure 6a, and it is associated with white noise.

**Figure 7:**
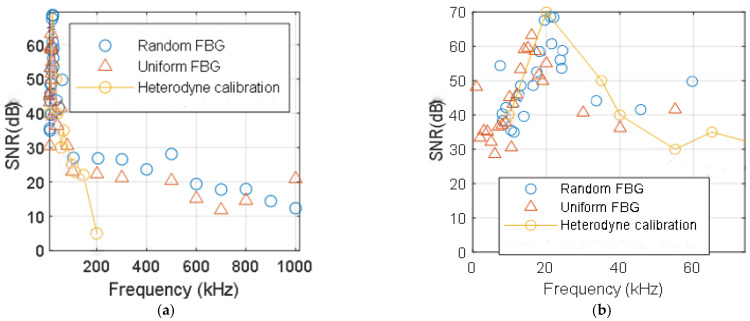
SNR of the random grating interrogation system and uniform FBG. (**a**) Whole spectra. (**b**) Detail of the resonance frequency of the PZT.

The SNR with respect to the frequency of the calibrating interferometer and the acousto-optic interrogator for random fiber gratings and uniform FBG is seen in Figure 7 for a 20 V sinusoidal signal. Figure 7a shows the whole spectrum, and Figure 7b shows the detail of the resonance frequency of the PZT. This point is equivalent to a 63 mrad or 10 nm peak displacement over 10.1 cm fiber length at 20 kHz (Figure 4a, A = 20 V).

Notably, the calibration interferometric system can detect amplitudes of 5 mV at 20 kHz applied to the PZT, equivalent to 35 μrad, approximately 57 pε of equivalent mechanical displacement measured at 1550 nm wavelength. Simultaneously, our acousto-optic interrogator can detect 25 mV of applied signal to the PZT at 20 kHz, which corresponds to 0.5 mrad in the calibration interferometer or 814 pε mechanical strain.

The acousto-optic interrogation of the random fiber grating accomplishes better frequency response than the interferometer, detecting frequencies up to 1 MHz. For frequencies far from the resonance frequency of the PZT and the aluminum plate, the SNR of the random fiber grating outperforms the SNR of the uniform FBG (Figure 7b). This improvement with respect to the uniform fiber Bragg grating is about 3 to 5 dB, depending on the frequency.

In the frequency domain, our system surpasses the interferometer, being able to reach 1 MHz of detectable surface acoustic waves, while the interferometer is only able to detect up to 200 kHz. For higher frequencies, the calibrating interferometer is unable to detect the strain applied to the fiber. However, our random fiber-grating-based system operates correctly up to 1 MHz of vibration signals.

**Figure 8:**
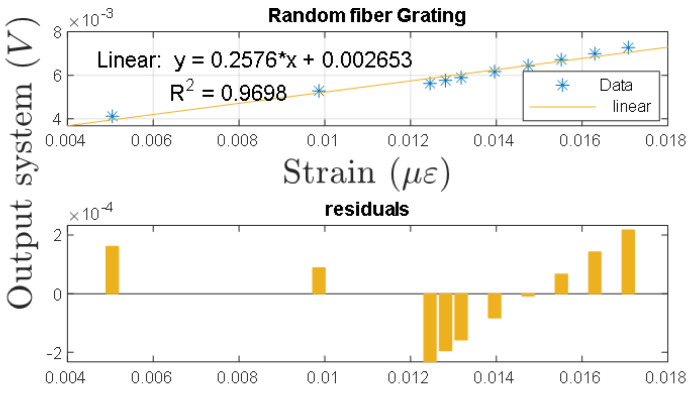
Response of the calibrating system to PZT strain excitation with output voltage.

Finally, Figure 8 shows the relationship of the output voltage of the random fiber grating system with respect to the calibrated strain applied. This characteristic shows the linear dependence between the strain and the output voltage of the random fiber grating over the observed small signal region.

## 4. Discussion

The ultimate displacement detection limit in the case of the calibrating interferometers and the grating readout system depends on the noise floor level. When the observed sideband harmonic falls below the noise floor, the system is incapable of detection because no value of amplitude can be used in the demodulation procedure. The measurement of the minimum detectable limit is carried out from the intermodal attenuation of the calibrating interferometer as shown in the Methods section and in [19]. When the first-order harmonic falls under the noise floor of the frequency domain, we can consider this point as a minimum detected amplitude. The noise level depends on the phase noise of the laser, the path imbalance of the interferometric systems, the bandwidth of measurement and the FFT parameters, especially the bandwidth measurement step. All these improvements lead to a good resolution for random fiber grating sensors. This fulfills the goal of high accuracy capabilities in the measurement. From [25], theoretical limits of around $10^{-9}$ radians can be achieved for bandwidths better than 1 Hz bandwidth and 100 mW optical power. In our case, due to white noise and wider bandwidth of the calibration interferometric system, we achieved a resolution better than $10^{-4}$ rad.

Another advantage of using random fiber grating is that there is no need to align the FBG Bragg wavelength with the laser source. This is very important, as random grating works at any wavelength. It also provides a better SNR over a medium-range frequency span (100–900 kHz) compared with the uniform random fiber grating.

Our approach is suitable to arbitrary reflection responses and arbitrary perturbations. It uses a comb with self-referenced calibration capability that extracts the slope between consecutive lines of the comb; therefore, it allows the demodulation of small signal perturbations with an arbitrary shape, which is a very powerful technique for small change detection.

## 5. Conclusions

In this paper, we show a novel approach to measuring vibrations with random fiber grating sensors and their calibration via interferometry techniques. The main system is based on amplitude modulation of a self-heterodyne acousto-optic comb that is injected into a random fiber grating. We calibrated the system with a heterodyne interferometer. The main system can detect ultrasonic and acoustic vibrations propagated along metal surfaces.

This system outperforms our previous work in strain accuracy and frequency [12] of multimode source implementation for grating sensor readout. This kind of system can be used, for example, for transformer integrity measurements as in [26,27]. Another important application is photoacoustic-microphone-based approaches for identifying compound concentrations as in [28], where the noise floor level over a time of 30 s and 1 Hz bandwidth is $2.45\times 10^{-8}\;$V RMS, which is in the same order as the results presented in this paper.

The system was tested with uniform FBG sensors as well as random fiber grating sensors. We demonstrated the initial hypothesis of system linearity, and we reached a very good resolution of 114 pε of peak-to-peak mechanical strain (or 57 pε of amplitude) of minimum detected signal.

## Data Availability

The data presented in this study are available on request from the corresponding author.
